# Association of severe COVID-19 outcomes with radiological scoring and cardiomegaly: findings from the COVID-19 inpatients database, Japan

**DOI:** 10.1007/s11604-022-01300-2

**Published:** 2022-07-26

**Authors:** Atsuhiro Kanayama, Yuuki Tsuchihashi, Yoichi Otomi, Hideaki Enomoto, Yuzo Arima, Takuri Takahashi, Yusuke Kobayashi, Koki Kaku, Tomimasa Sunagawa, Motoi Suzuki, The COVID-19 discharge summary database (CDSD) group, Yusuke Ajishi, Hiroshi Ishii, Satoru Ishikawa, Hajime Iwagoe, Yasushi Kaneko, Kei Kasahara, Yoji Kawaguchi, Masafumi Masuda, Momoko Mawatari, Yasunori Mishima, Yoji Nagasaki, Norio Ohmagari, Kensaku Okada, Hiroshi Satoh, Yasuhiko Terai, Katsuya Tsujie, Haruhito Watase

**Affiliations:** 1 Center for Field Epidemic Intelligence, Research, and Professional Development, National Institute of Infectious Diseases, 1-23-1 Toyama, Shinjuku-ku, Tokyo, 162-8640 Japan; 2 Division of Infectious Diseases Epidemiology and Control, National Defense Medical College Research Institute, 3-2 Namiki, Tokorozawa, Saitama 359-8513 Japan; 3 Center for Surveillance, Immunization, and Epidemiologic Research, National Institute of Infectious Diseases, 1-23-1 Toyama, Shinjuku-ku, Tokyo, 162-8640 Japan; 4 Department of Radiology, Tokushima University Hospital, 2-50-1 Kuramoto, Tokushima-city, Tokushima 770-8503 Japan

**Keywords:** Chest X-ray scores, Chest CT scores, COVID-19, Cardiomegaly, Severity

## Abstract

**Purpose:**

We aimed to characterize novel coronavirus infections based on imaging [chest X-ray and chest computed tomography (CT)] at the time of admission.

**Materials and methods:**

We extracted data from 396 patients with laboratory-confirmed COVID-19 who were managed at 68 hospitals in Japan from January 25 to September 2, 2020. Case patients were categorized as severe (death or treatment with invasive ventilation during hospitalization) and non-severe groups. The imaging findings of the groups were compared by calculating odds ratios (ORs) and 95% confidence intervals (95% CIs), adjusted for sex, age, and hospital size (and radiographic patient positioning for cardiomegaly). Chest X-ray and CT scores ranged from 0 to 72 and 0 to 20, respectively. Optimal cut-off values for these scores were determined by a receiver-operating characteristic (ROC) curve analysis.

**Results:**

The median age of the 396 patients was 48 years (interquartile range 28–65) and 211 (53.3%) patients were male. Thirty-two severe cases were compared to 364 non-severe cases. At the time of admission, abnormal lesions on chest X-ray and CT were mainly observed in the lower zone/lobe. Among severe cases, abnormal lesions were also seen in the upper zone/lobe. After adjustment, the total chest X-ray and CT score values showed a dose-dependent association with severe disease. For chest X-ray scores, the area under the ROC curve (AUC) was 0.91 (95% CI = 0.86–0.97) and an optimal cut-off value of 9 points predicted severe disease with 83.3% sensitivity and 84.7% specificity. For chest CT scores, the AUC was 0.94 (95% CI = 0.89–0.98) and an optimal cut-off value of 11 points predicted severe disease with 90.9% sensitivity and 82.2% specificity. Cardiomegaly was strongly associated with severe disease [adjusted OR = 24.6 (95% CI = 3.7–166.0)].

**Conclusion:**

Chest CT and X-ray scores and the identification of cardiomegaly could be useful for classifying severe COVID-19 on admission.

**Supplementary Information:**

The online version contains supplementary material available at 10.1007/s11604-022-01300-2.

## Introduction

Severe acute respiratory syndrome coronavirus 2 (SARS-CoV-2) infections began to spread worldwide in 2020 [1, 2]. In Japan, the number of notifications in relation to disease caused by SARS-CoV-2 (COVID-19) reached its first peak in April 2020 and its second peak in August 2020 [3, 4]. The deterioration of COVID-19 inpatients, suggested by the use of ventilation equipment, is a burden for both inpatients and facilities with limited medical resources. Many factors are suggested to predict a severe outcome [5, 6]. In addition to demographics, signs/symptoms that appear prior to admission, and hematological/biochemical laboratory findings, imaging analyses could be powerful tools for predicting the prognosis of COVID-19 patients at an early stage. The most common chest computed tomography (CT) patterns are ground-glass opacities (GGOs) and bilateral consolidation in the peripheral lower lung fields [7, 8]. CT seems to have superior diagnostic accuracy to chest X-ray [9]; however, in comparison to CT, chest X-ray may have advantages in terms of portability, accessibility, radiation exposure, and cost, and could provide benefits to both healthcare providers and patients. Therefore, we aimed to characterize COVID-19 inpatients based on chest X-ray findings in comparison to CT findings that were stored in a database that was established in Japan in February 2020.

## Methods

### Data collection

COVID-19 became a notifiable disease under the Infectious Diseases Control Law on February 1, 2020. On February 20, 2020, the Ministry of Health, Labour and Welfare of Japan requested that public health centers (PHCs) collect the discharge summary of each COVID-19 patient from hospital and to report it to the National Institute of Infectious Diseases (NIID) under the Infectious Diseases Control Law. The summary included the following data: demographics, date of onset, hospitalization, and discharge, medical history before hospitalization, clinical course during hospitalization, hematological, biochemical, and radiological imaging test results at the time of admission, treatments during hospitalization, and the outcome on discharge. As of September 14, 2020, a total of 396 reports were collected from 68 hospitals and registered in the database for analysis. Hematological, biochemical, and radiological imaging tests on admission were defined as tests performed from 3 days before to 3 days after admission. A severe case was defined as an inpatient who died or who received mechanical ventilation; cases in which an inpatient did not die or receive mechanical ventilation were defined as non-severe.

### Imaging analysis

All images were interpreted independently by two radiologists with 18 years (Y.O.) and 4 years (H.E.) of experience, respectively, in clinical radiology. CT images were evaluated for the following: lesion distribution (single/both lungs, central/peripheral), presence of GGOs, consolidation, air bronchogram, crazy paving pattern, subpleural line, bronchial wall thickening, bronchiectasis, pleural thickening, cavitation, CT halo, reversed halo, pleural effusion, pericardial effusion, pulmonary emphysema, pulmonary fibrosis, and hilar and/or mediastinal lymphadenopathy. In addition, each lobe of the lung was assessed for opacification, and the lesion size was graded as follows: 0 (none), 1 (< 1 cm), 2 (1–3 cm), 3 (3 cm to < 50% of the lobe), or 4 (50–100% of the lobe). The score for each lobe was summed to produce a total score ranging from 0 to 20 [10, 11]. This total score was categorized into three groups: (1) ≤ 10 points, (2) 11–15 points, and (3) 16–20 points.

Chest X-ray images were evaluated for the following: reticular-nodular opacities, consolidation, cardiomegaly, nodules, pleural effusion, pneumothorax, and distribution (peripheral/perihilar/diffuse/basal predominance/superior predominance). The lungs were divided into six zones (right upper, right middle, right lower, left upper, left middle, and left lower), with each zone spanning one-third of the craniocaudal distance of the lung field [12]. The extent of airspace opacity in each zone was scored (0, no opacity; 1, < 25% opacity; 2, 25–49% opacity; 3, 50–74% opacity; 4, ≥ 75% opacity). The density of opacities was scored [0, clear; 1, hazy (vessel markings clearly visible); 2, moderate (vessel markings are partially obscured); 3, dense (vessels obscured, air bronchogram may be present)] according to the modified RALE score [12, 13]. The extent of involvement was calculated by multiplying the opacity by the density, yielding a score per zone ranging from 0 to 12. The total score for six zones ranged from 0 to 72. This total score was categorized into four groups: (1) ≤ 6 points; (2) 7–12 points; (3) 13–18 points; and (4) 19 points and over. A cardiothoracic ratio (CTR) of > 50% on an upright posterior–anterior (PA) chest X-ray and a CTR of > 55% on a decubitus or sitting anterior–posterior (AP) chest X-ray were considered abnormal and suggestive of cardiomegaly [14].

### Statistical analyses

We described cases according to the demographics, symptoms, serological, and radiological test results at the time of admission. Odds ratios (ORs) and 95% confidence intervals (95% CIs) were calculated using logistic regression to assess the association between severe disease status and chest CT/X-ray score; adjusted ORs controlling for age, sex, and hospital size (dichotomized into two groups of < 600 beds and ≥ 600 beds) were also calculated (further adjustment for X-ray radiographic patient positioning (PA or AP) was done for cardiomegaly). The *p* values for the serological test results were calculated by Wilcoxon rank sum test. A receiver-operating characteristic (ROC) curve analysis was conducted to calculate the area under the ROC curve (AUC) with 95% CI to estimate the optimal cut-off value of the chest CT/X-ray score. All analyses were conducted using STATA (ver. 16, Stata Corp, USA).

### Ethics

The current study was conducted under Articles 12 and 15 of the Infectious Diseases Control Law in Japan and was therefore exempt from the requirement for ethical review, including the requirement of informed consent. The analysis was performed for public health purposes and none of the data presented included identifiable information.

## Results

Three hundred ninety-six patients who were hospitalized from January 25, 2020 to September 2, 2020 were included in the present study. The patients were discharged after a median period of 13 days (IQR 8–19). The median age of the patients was 48 years (IQR 28–65), 211 (53.3%) were males, 118 (30.0%) had comorbidities, and 33 (8.3%) were smokers. These cases were classified into the severe group (*n* = 32) and the non-severe group (*n* = 364). The median time from the onset to hospitalization was 4.5 days (IQR 2.0–9.0) in the severe group and 5.0 days (IQR 2.0–8.0) in the non-severe group. A large proportion of the patients in the severe group were male and elderly (Table 1). Older age [OR = 1.10 (95% CI = 1.07–1.13) per increase in year] and male sex [OR = 2.8 (1.2–6.5)] were associated with severe disease. Patients with severe disease tended to have underlying diseases [e.g., hypertension, OR = 5.0 (95% CI = 2.3–10.7); diabetes, 6.2 (2.8–13.9); or malignant tumor, 23.9 (7.3–78.8)]. Regarding cancer, the severe group included 3 patients with prostate cancer, 1 patient with gastric cancer, and 1 patient with a thyroid tumor. In contrast, the non-severe group included 1 patient with breast cancer, 1 patient with cervical cancer, 1 patient with prostate cancer, 1 patient with colon cancer, and 1 patient with malignant lymphoma. A larger proportion of patients in the severe group had fever and respiratory symptoms from the onset of disease to admission in comparison to the non-severe group (Table 2). Few of the patients in the severe group had dysgeusia or dysosmia. Overall, 58 (13.9%) patients were asymptomatic both on admission and before admission. As for serological test results (Table 3), in severe cases, elevated levels of lactate dehydrogenase (LDH) and C-reactive protein (CRP) were observed and were higher in severe cases (*p* < 0.01 per test).

**Table 1:** Demographic characteristics of COVID-19 inpatients at the time of admission

	Severe (*n* = 32)		Non-severe (*n* = 364)	
	*n*	(%)	*n*	(%)
Sex (male)	24	(75.0)	187	(51.4)
Age	75	(70–85)	45	(27–60)
Any underlying disease	23	(71.9)	95	(26.1)
Hypertension	14	(43.8)	49	(13.5)
Diabetes	12	(37.5)	32	(8.8)
Malignant tumor	8	(25.0)	5	(1.4)
Dyslipidemia	5	(15.6)	33	(9.1)
COPD	4	(12.5)	2	(0.5)
Cerebrovascular disease	3	(9.4)	2	(0.5)
Cardiovascular disease	1	(3.1)	3	(0.8)
Smoker	2	(6.3)	31	(8.5)

Age is presented as the median and interquartile range

*COPD* chronic obstructive pulmonary disease

**Table 2:** Symptoms of COVID-19 inpatients at the time of admission

	Severe (*n* = 32)		Non-severe (*n* = 364)	
	*n*	(%)	*n*	(%)
Fever	27	(84.4)	232	(63.7)
Respiratory symptom(s)	21	(65.6)	194	(53.3)
Dyspnea	17	(53.1)	59	(16.2)
Cough	11	(34.4)	133	(36.5)
Pharyngeal pain	3	(9.4)	77	(21.2)
Gastrointestinal symptom(s)	9	(28.1)	63	(17.3)
Anorexia	6	(18.8)	36	(9.9)
Diarrhea	2	(6.3)	32	(8.8)
Nausea and vomiting	2	(6.3)	9	(2.5)
Abdominal pain	0	(0.0)	5	(1.4)
Malaise	8	(25.0)	83	(22.8)
Nasal discharge	2	(6.3)	30	(8.2)
Headache	1	(3.1)	57	(15.7)
Consciousness disorder	1	(3.1)	0	(0.0)
Dysgeusia	1	(3.1)	67	(18.4)
Dysosmia	0	(0.0)	64	(17.6)
Arthralgia	0	(0.0)	22	(6.0)
Myalgia	0	(0.0)	10	(2.7)
No signs or symptoms	1	(3.1)	57	(15.7)

**Table 3:** Serological test results of COVID-19 inpatients at the time of admission

	Severe (*n* = 32)			Non-severe (*n* = 364)			
	*n*	Median	(IQR)	*n*	Median	(IQR)	
WBCs (/μL)	23	7080	(4785–8435)	303	5000	(4055–6200)	
Neutrophil (/μL)	18	5351	(4270–7303)	278	3023	(2132–3918)	
Lymphocyte (/μL)	18	822	(651–1553)	289	1312	(1003–1750)	
RBCs (10^4^/μL)	19	389	(306–462)	286	476	(438–513)	
Plt (10^4^/μL)	23	16.5	(12.3–22.2)	296	24.0	(18.3–78.0)	
AST (IU/L)	22	46	(29–67)	297	23	(19–31)	
ALT (IU/L)	21	29	(19–32)	297	20	(14–32)	
LDH (IU/L)	22	348	(315–544)	290	183	(158–224)	
BUN (mg/dL)	19	23.3	(18.3–44.5)	279	11.9	(9.6–14.5)	
Cre (mg/dL)	19	1.2	(0.8–1.4)	274	0.76	(0.6–0.9)	
CRP (mg/dL)	21	9.8	(4.4–14.5)	291	0.4	(0.1–1.8)	

*IQR* interquartile range, *WBC* white blood cell, *RBC* red blood cell, *Plt* platelet, *AST* aspartate aminotransferase, *ALT* alanine aminotransferase, *LDH* lactate dehydrogenase, *BUN* blood urea nitrogen, *Cre* creatinine, *CRP* C-reactive protein

**Fig. 1:**
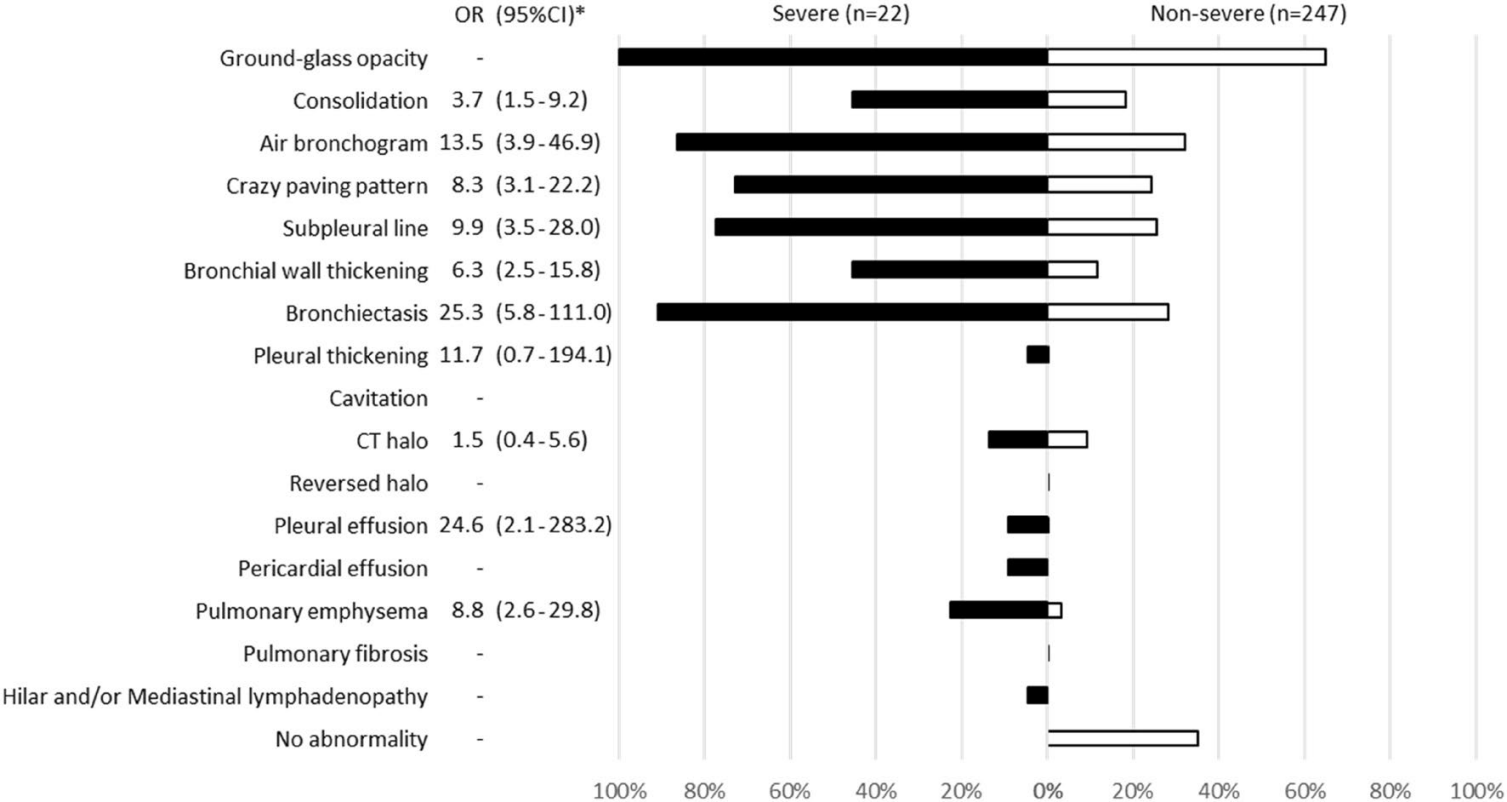
Chest CT findings at the time of admission. The proportion of cases with each finding is shown with black (severe) and white (non-severe) bars. *ORs and 95% CIs for Yes vs. No for each abnormality. *OR* odds ratio, *95% CI* 95% confidence interval

**Fig. 2:**
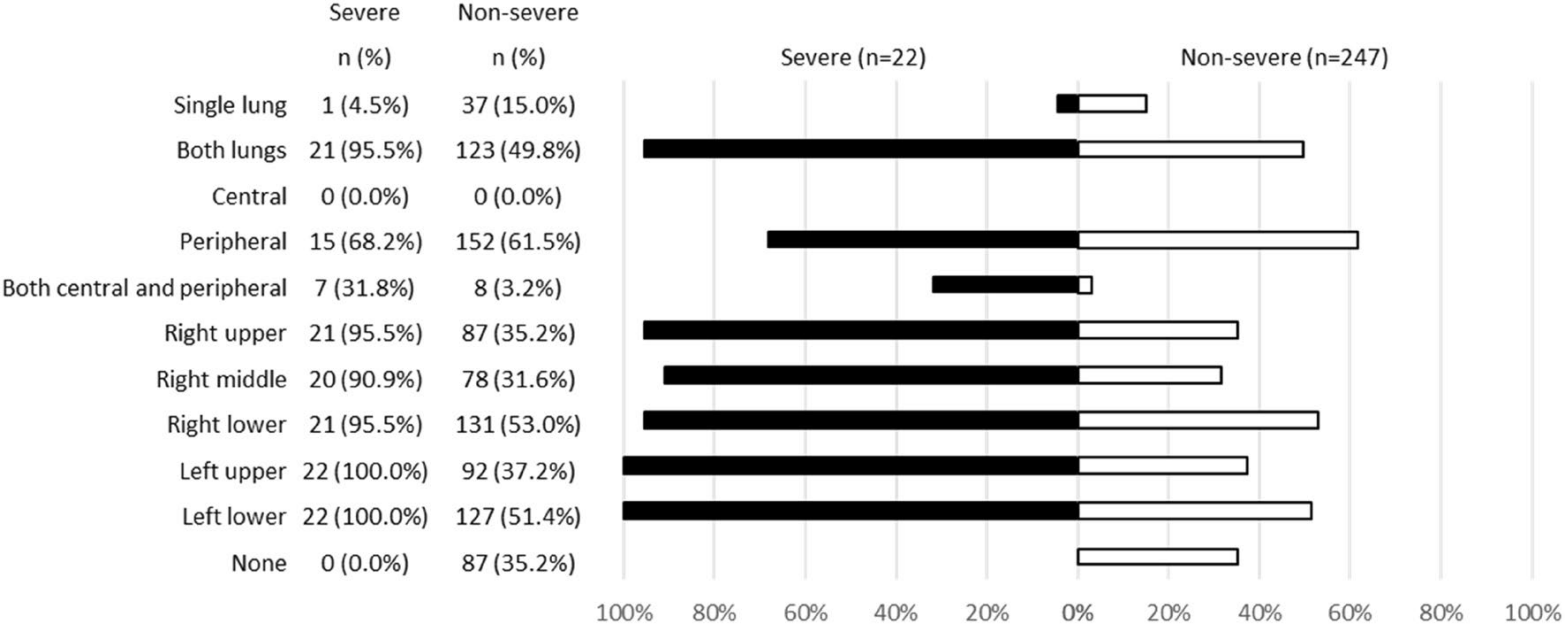
Distribution of chest CT abnormalities at the time of admission. The proportion of cases with each distribution is shown by black (severe) and white (non-severe) bars

**Fig. 3:**
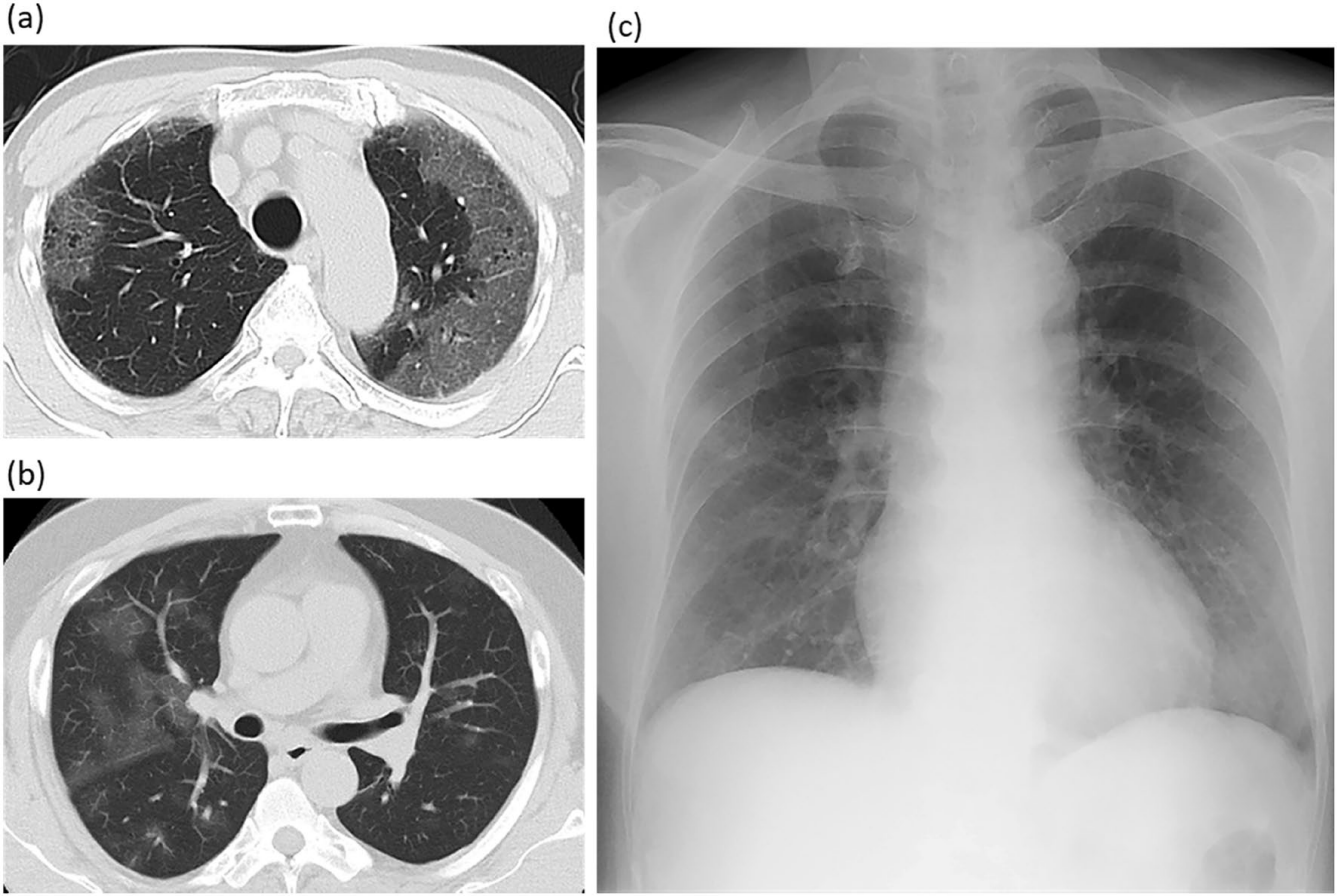
COVID-19 chest CT and X-ray images on admission. **a** Chest CT image of a 68-year-old male patient with ground-glass opacities (GGO) in the peripheral region (non-severe case). **b** Chest CT image of a 51-year-old male patient with GGO in the peripheral and central regions (non-severe case). **c** Anterior–posterior (AP) chest X-ray image of a 70-year-old female patient with cardiomegaly (severe case)

**Fig. 4:**
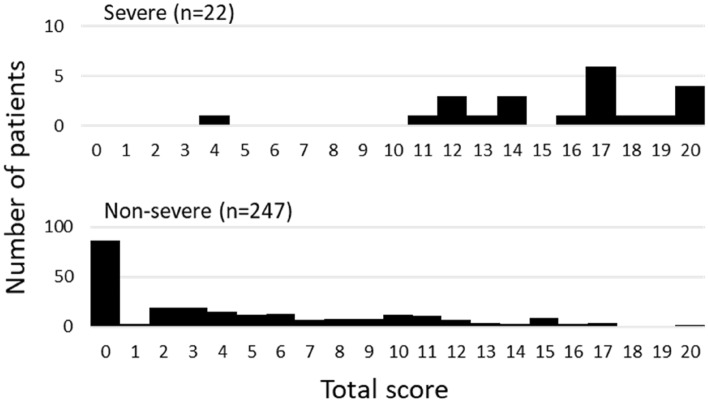
Total chest CT score distribution at the time of admission among severe and non-severe patients. Number of severe (top) or non-severe (bottom) patients per total chest CT score on admission is shown

Among 269 patients for whom CT images obtained at the time of admission were available, the most frequent findings in severe cases were GGO, followed by bronchiectasis, air bronchogram, subpleural line, crazy paving pattern, consolidation, and bronchial wall thickening (Fig. 1). Abnormalities were distributed in the peripheral region, but in severe cases, they also spread to the central region, and in most cases, this was observed bilaterally (Fig. 2). While one case had pulmonary edema and another had aspiration pneumonia, all cases had lesions due to COVID-19 pneumonia; Fig. 3 shows two representative cases with such lesions on CT. In non-severe cases, GGO was observed in the peripheral region (Fig. 3a), but was also found in the central region in some patients (Fig. 3b). Elevated total scores were observed in severe cases (Fig. 4; see also Supplementary Table 1). Severe cases presented high scores in not only the lower lobes but also the upper lobes (Supplementary Table 1). The total score groups were associated with severe disease in a dose-dependent manner as shown by the increase in the crude ORs with increase in total score (Table 4, top). The OR adjusted for sex and age (aOR) showed similar results; adjusted for sex, age, and hospital size, the associations of the CT scores remained the same. In addition, when the total score was treated as a continuous rather than categorical variable, the OR increased by 1.5 (95% CI = 1.3–1.7) for every 1-point increase in total score, and increased by 1.4 (95% CI = 1.2–1.6) when adjusted for age, sex, and hospital size. In the ROC analysis of the total score, the AUC was 0.93 (95% CI = 0.89–0.98) and an optimal cut-off score of 11 was found to predict severe disease with 90.9% sensitivity and 82.2% specificity.

**Table 4:** Association between radiological scores at the time of admission and severe outcomes

Chest CT	Severe (*n* = 22)	Non-severe (*n* = 247)		Crude		Adjusted for sex and age*		Adjusted for sex, age, and hospital size*
Total score	*n* (%)	*n* (%)		OR (95% CI)		aOR (95% CI)		aOR (95% CI)
0–10	1 (4.5)	203 (82.2)		Reference				
11–15	8 (36.4)	34 (13.8)		47.8 (5.8–394.1)		8.7 (3.4–22.0)		8.7 (3.4–22.1)
16–20	13 (59.1)	10 (4.0)		263.9 (31.3–2222.3)				

Chest X-ray	Severe (*n* = 18)	Non-severe (*n* = 150)		Crude		Adjusted for sex and age*		Adjusted for sex, age, and hospital size*
Total score	*n* (%)	*n* (%)		OR (95% CI)		aOR (95% CI)		aOR (95% CI)
0–6	3 (16.7)	124 (82.7)		Reference				
7–12	3 (16.7)	12 (8.0)		10.3 (1.9–56.9)		2.2 (1.3–3.7)		2.1 (1.2–3.6)
13–18	2 (11.1)	5 (3.3)		16.5 (2.2–122.2)				
19–72	10 (55.6)	9 (6.0)		45.9 (10.7–197.1)				

*CT* computed tomography, *OR* odds ratio, *aOR* adjusted OR, *95% CI* 95% confidence interval

^*^Increase per total score group (0–10, 11–15, and 16–20 for chest CT and 0–6, 7–12, 13–18, and 19–72 for chest X-ray), based on the results of the crude analysis. Hospital size dichotomized into two groups of < 600 beds and ≥ 600 beds

**Fig. 5:**
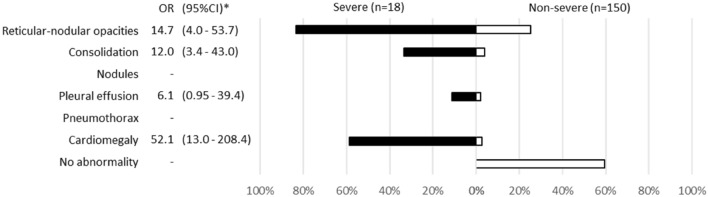
Chest X-ray findings at the time of admission. The proportion of cases with each finding is shown with black (severe) and white (non-severe) bars. Only for cardiomegaly, the severe case group consisted of 17 patients. *ORs and 95% CIs for Yes vs. No for each abnormality. *OR* odds ratio, *95% CI* 95% confidence interval

**Fig. 6:**
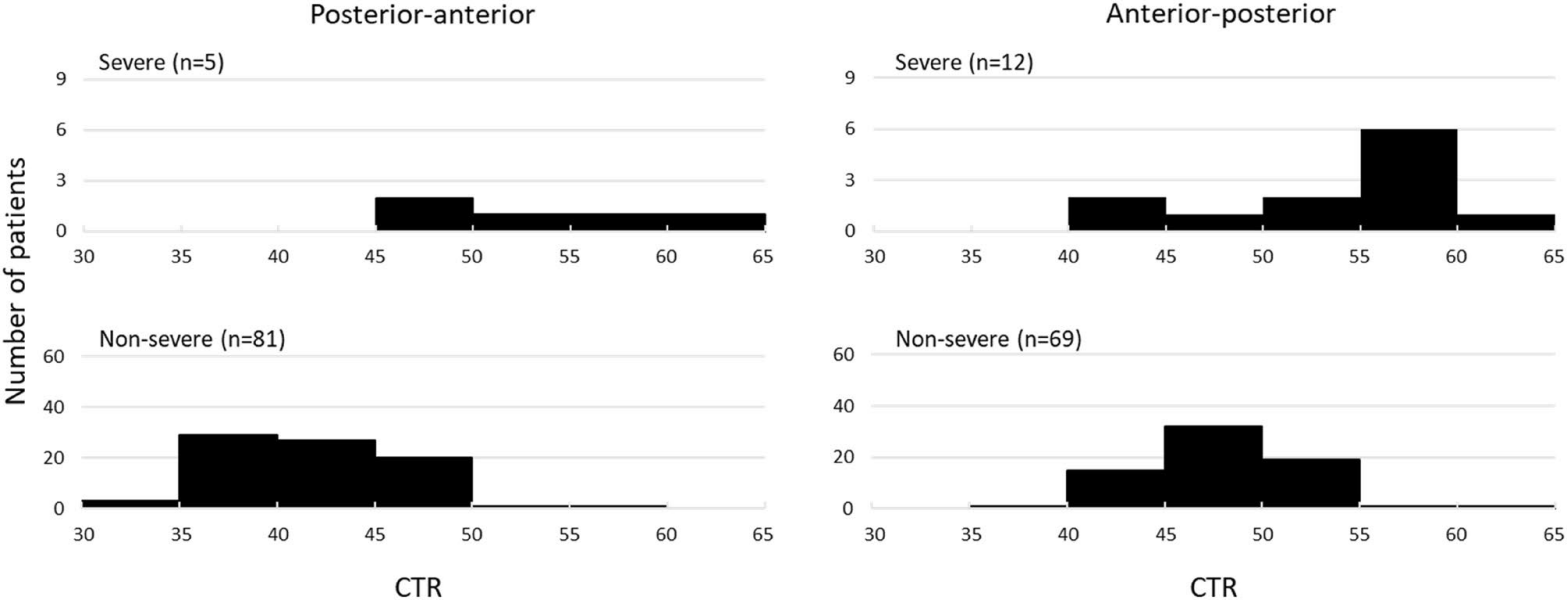
Cardiothoracic ratio distribution by radiographic patient positioning among severe and non-severe patients. The number of severe (top) or non-severe (bottom) patients per 5 points width of cardiothoracic ratio (CTR) in the posterior–anterior (left) or anterior–posterior (right) chest X-ray image on admission is shown

**Fig. 7:**
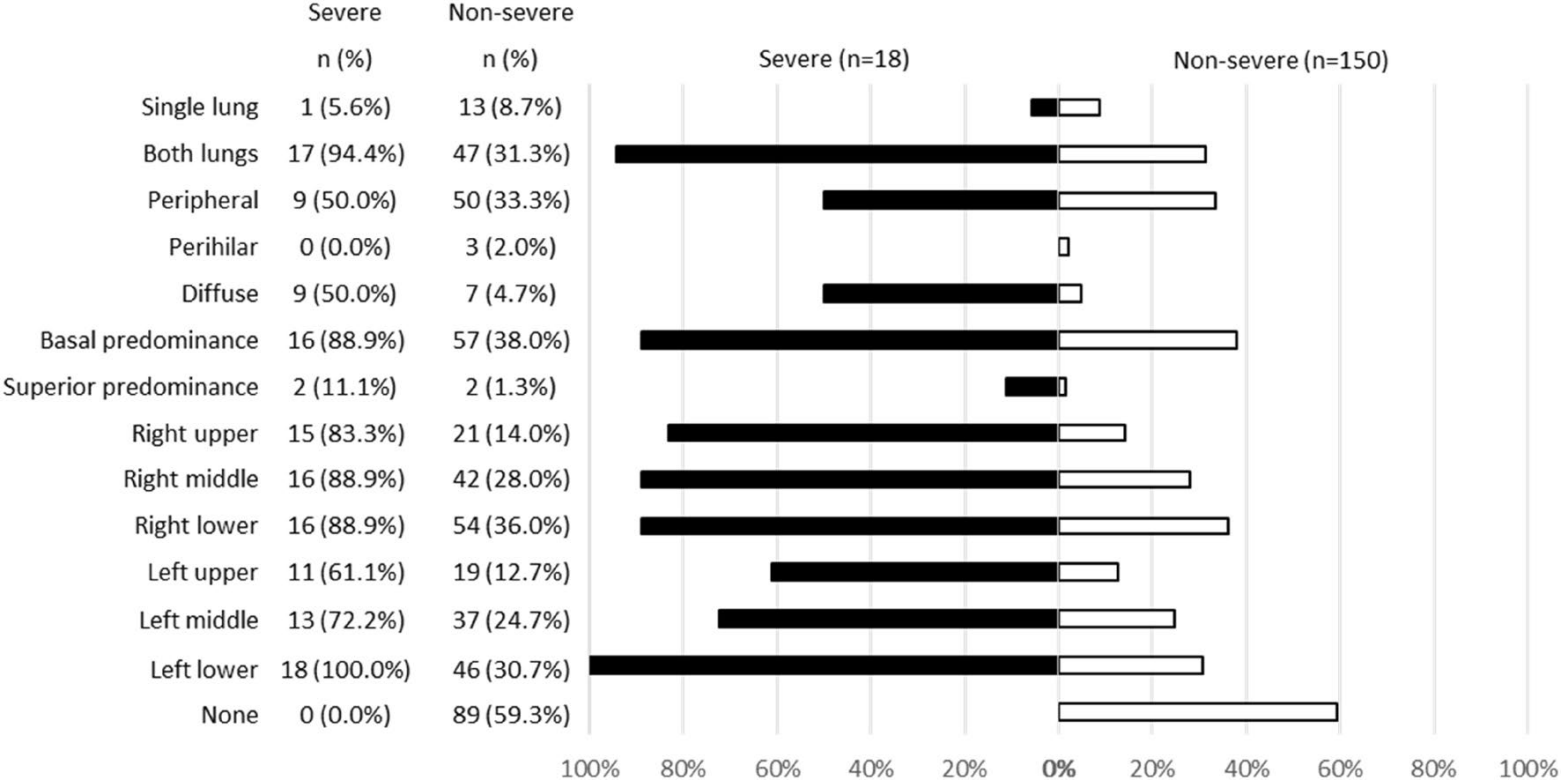
Distribution of abnormalities in chest X-ray at the time of admission. The proportion of cases with each distribution is shown by black (severe) and white (non-severe) bars

**Fig. 8:**
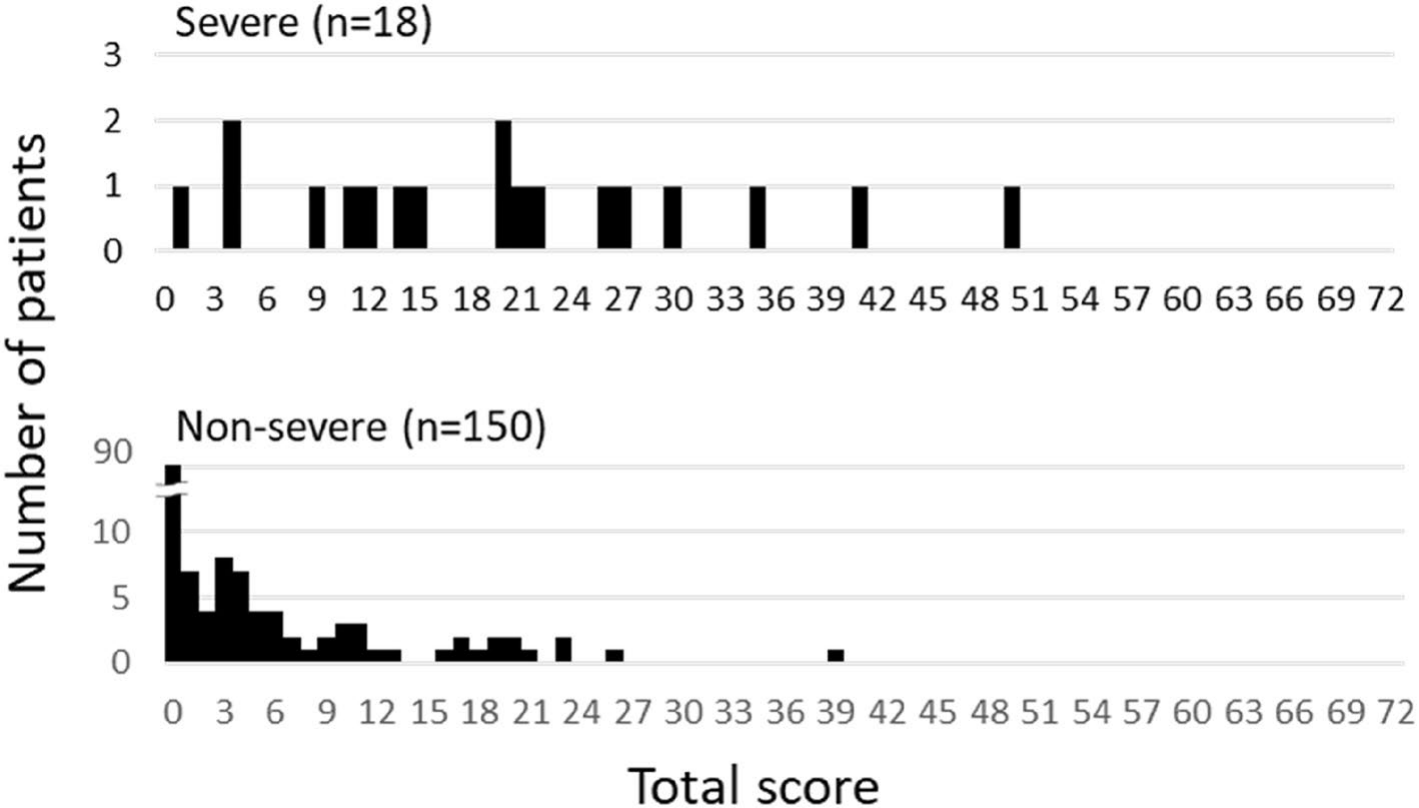
Total chest X-ray score distribution at the time of admission among severe and non-severe patients. Number of severe (top) or non-severe (bottom) patients per total chest X-ray score on admission is shown

Among 168 cases for which chest X-ray images obtained at the time of admission were available, the common findings of severe cases included reticular-nodular opacities, cardiomegaly, and consolidation (Fig. 3c and Fig. 5). In particular, high CTR values were observed in both PA and AP views of severe cases (Fig. 6) and cardiomegaly was strongly associated with severe disease [OR = 52.1 (95% CI = 13.0–208.4); aOR for sex, age, hospital size, and patient positioning of chest X-ray = 24.6 (95% CI = 3.7–166.0)]. For cardiomegaly, a case was excluded from the calculation due to insufficient information to classify to PA or AP. Abnormalities were distributed in both lungs, in each zone, and showed a basal predominance (Fig. 7; see also Supplementary Table 2). High total scores were also observed in severe cases (Fig. 8; see also Supplementary Table 2). The total score groups were associated with severe disease in a dose-dependent manner (Table 4, bottom, crude OR), and the aOR controlling for sex and age showed similar results. In addition, adjusted for hospital size, the association remained similar between severe disease and chest X-ray score. When the total score was treated as a continuous rather than categorical variable, the OR increased by 1.2 (95% CI = 1.1–1.2) for every 1-point increase in total score, and the aOR increased by 1.1 (95% CI = 1.0–1.2), after adjustment for sex, age, and hospital size. An ROC analysis of the total score revealed that the AUC was 0.91 (95% CI = 0.86–0.97) and an optimal cut-off value of 9 predicted severe disease with 83.3% sensitivity and 84.7% specificity. Considering the presence of cardiomegaly on admission, for patients without cardiomegaly on admission, a high chest X-ray score of ≥ 9 showed a strong association with severe disease (crude OR = 44.1; 95% CI = 5.1–377.7; aOR controlling for sex, age, and hospital size = 11.7; 95% CI = 1.1–119.5). In patients with cardiomegaly on admission, a high chest X-ray score was not associated with severe disease (crude OR = 1.3; 95% CI = 0.1–20.7).

## Discussion

This is the first comprehensive report on the characteristics of the imaging findings on admission among laboratory-confirmed COIVD-19 patients in Japan. Older age and male sex were associated with severe disease, which was consistent with previous reports [1, 15, 16]. Abnormal signs in severe cases were mostly observed in the peripheral and basal regions; in severe cases, abnormalities were more frequently observed in the central region of both lungs. In logistic regression, adjusted for sex, age, and hospital size, a dose-dependent association between severe disease and the total chest X-ray and CT scores was found. Regarding the chest X-ray score, an optimal cut-off value of 9 points was found to predict severe disease with high sensitivity and specificity, although the sensitivity was lower than that of the CT score. Of note, adjusted for sex, age, hospital size, and radiographic patient positioning, cardiomegaly was strongly associated with severe disease.

In the severe group, GGO was observed on CT in all cases, with most showing progress to the crazy paving pattern, air bronchogram, and bronchiectasis. The lesions of severe COVID pneumonia are reported to be mainly located in the lower lobe/zone but not in the upper lobe/zone [5, 7, 10, 17]; however, in the severe cases in our study, the lesions of COVID pneumonia had already spread evenly to the upper lobes/zones by the time of admission (Supplementary Tables 1 and 2). Our analysis strongly suggests that the chest X-ray and CT scores, including for the upper lobes/zones, are useful as an indicator of severity. The time period from the onset to hospitalization of the severe cases (4.5 days) was similar to that of the non-severe cases (5 days); thus, the reason for the development of lesions in the upper lobe/zone in the severe cases may not be delayed hospitalization and remains unclear.

To the best of our knowledge, this is the first report to link cardiomegaly to severity on chest X-ray images. In this study, there was no history of heart disease as an underlying disease in any of the cases with cardiomegaly. The reason why cardiomegaly was observed on chest X-ray images may be due to prior hypertension and/or the right-ventricular overload caused by COVID-19-induced pulmonary hypertension [18]. Multiple mechanisms have been suggested to underlie the development of pulmonary hypertension, including worsening myocardial injury, cytokine storm, and the presence of thrombotic microangiopathy [19]. On the other hand, a report of three administrative autopsies of undiagnosed COVID-19-related deaths identified left-ventricular hypertrophy in each autopsy and all cases had prior hypertension [20]. We confirmed that 15 of 17 cases with cardiomegaly in the severe group were not in a severe state (i.e., they did not require mechanical ventilation) on admission; the remaining 2 cases with cardiomegaly were in a severe state (i.e., they received mechanical ventilation) on admission and showed high chest X-ray scores. Overall, these findings indicate that the identification of patients with cardiomegaly on admission may be useful for the classification of severe disease.

Searching for cardiomegaly on chest X-ray images will be even more noteworthy when CT examinations are difficult to perform for some reason (e.g., when there are restrictions in relation to bringing patients into the CT laboratory, or when there is a shortage of CT equipment). With regard to other relevant chest X-ray findings, we identified that high scores of both airspace opacity and density in the upper and middle zones could be useful when considering severity. Furthermore, we identified an optimal cut-off value of 9, which showed 83.3% sensitivity and 84.7% specificity. The specificity was a little higher than that of CT (82.2%).

The present study was associated with some limitations. Data were not systematically collected and it is possible that the COVID-19 inpatients included asymptomatic patients who were hospitalized as an administrative response. In consideration of this potential bias, a sensitivity analysis was performed with the sample restricted to symptomatic subjects; however, there was little change in the association between severe disease and the CT score [aOR = 7.8 (95% CI = 3.1–19.9)] or chest X-ray score [aOR = 2.0 (1.1–3.4)], and the aOR for cardiomegaly on chest X-ray remained high [23.3 (3.3–165.1)] after adjustment for sex, age, hospital size, and radiographic patient positioning. Cardiomegaly, indeed, may be involved in severe outcomes. However, the patient data on BMI, obesity, and the presence of cardiomegaly prior to SARS-CoV-2 infection were insufficient, and further investigation is required.

In conclusion, we characterized the imaging findings of COVID-19 inpatients on admission. We emphasize that the use of chest X-ray for scoring and identifying cardiomegaly is useful for the classification of severe COVID-19 on admission.

## Supplementary Information

Below is the link to the electronic supplementary material.

Supplementary file1 (DOCX 35 KB)
