# Supplementary material for: Association of severe COVID-19 outcomes with radiological scoring and cardiomegaly: findings from the COVID-19 inpatients database, Japan
Source: Jpn J Radiol. 2022 Jul 26;40(11):1138–47. doi: 10.1007/s11604-022-01300-2 (PMC9315080; doi:10.1007/s11604-022-01300-2)
Supplement: Supplementary file 1 — Supplementary file1 (DOCX 35 KB) [file 11604_2022_1300_MOESM1_ESM.docx]

**Supplementary Table 1. Chest CT scores for each lobe in severe and non-severe patients**

|  |  | **Severe (n=22)** | | |  | **Non-severe (n=247)** | | |
| --- | --- | --- | --- | --- | --- | --- | --- | --- |
| **Lobe Lesion size** | **Score** | **n** |  | **%** |  | **n** |  | **%** |
| Right Upper 0 | 0 | 1 |  | 4.5 |  | 160 |  | 64.8 |
| >0 and <1 cm | 1 | 0 |  | 0.0 |  | 17 |  | 6.9 |
| 1 cm to <3 cm | 2 | 3 |  | 13.6 |  | 34 |  | 13.8 |
| 3cm to <50% lobe | 3 | 8 |  | 36.4 |  | 31 |  | 12.6 |
| ≥50% lobe | 4 | 10 |  | 45.5 |  | 5 |  | 2.0 |
| Right Middle 0 | 0 | 2 |  | 9.1 |  | 169 |  | 68.4 |
| >0 and <1 cm | 1 | 0 |  | 0.0 |  | 24 |  | 9.7 |
| 1 cm to <3 cm | 2 | 7 |  | 31.8 |  | 32 |  | 13.0 |
| 3 cm to <50% lobe | 3 | 6 |  | 27.3 |  | 20 |  | 8.1 |
| ≥50% lobe | 4 | 7 |  | 31.8 |  | 2 |  | 0.8 |
| Right Lower 0 | 0 | 1 |  | 4.5 |  | 116 |  | 47.0 |
| >0 and <1 cm | 1 | 0 |  | 0.0 |  | 12 |  | 4.9 |
| 1 cm to <3 cm | 2 | 1 |  | 4.5 |  | 39 |  | 15.8 |
| 3 cm to <50% lobe | 3 | 10 |  | 45.5 |  | 69 |  | 27.9 |
| ≥50% lobe | 4 | 10 |  | 45.5 |  | 11 |  | 4.5 |
| Left Upper 0 | 0 | 0 |  | 0.0 |  | 155 |  | 62.8 |
| >0 and <1 cm | 1 | 1 |  | 4.5 |  | 22 |  | 8.9 |
| 1 cm to <3 cm | 2 | 6 |  | 27.3 |  | 34 |  | 13.8 |
| 3 cm to <50% lobe | 3 | 8 |  | 36.4 |  | 32 |  | 13.0 |
| ≥50% lobe | 4 | 7 |  | 31.8 |  | 4 |  | 1.6 |
| Left Lower 0 | 0 | 0 |  | 0.0 |  | 120 |  | 48.6 |
| >0 and <1 cm | 1 | 0 |  | 0.0 |  | 15 |  | 6.1 |
| 1 cm to <3 cm | 2 | 2 |  | 9.1 |  | 49 |  | 19.8 |
| 3 cm to <50% lobe | 3 | 10 |  | 45.5 |  | 53 |  | 21.5 |
| ≥50% lobe | 4 | 10 |  | 45.5 |  | 10 |  | 4.0 |

**Supplementary Table 2. Radiological chest X-ray scores for each lung zone in severe and non-severe patients**

| **Airspace opacity** | |  | **Severe**  **n=18** | | |  | **Non-severe n=150** | | |  | **Density** | |  | **Severe**  **n=18** | | |  | **Non-severe**  **n=150** | | |
| --- | --- | --- | --- | --- | --- | --- | --- | --- | --- | --- | --- | --- | --- | --- | --- | --- | --- | --- | --- | --- |
| **Lung zone** | **Opacity (%)** | **Score** | **n** |  | **%** |  | **n** |  | **%** |  | **Lung zone** | **Density** | **Score** | **n** |  | **%** |  | **n** |  | **%** |
| Right Upper | 0 | 0 | 3 |  | 16.7 |  | 129 |  | 86.0 |  | Right Upper | clear | 0 | 3 |  | 16.7 |  | 129 |  | 86.0 |
|  | <25 | 1 | 6 |  | 33.3 |  | 12 |  | 8.0 |  |  | hazy | 1 | 12 |  | 66.7 |  | 18 |  | 12.0 |
|  | 25-49 | 2 | 2 |  | 11.1 |  | 5 |  | 3.3 |  |  | moderate | 2 | 3 |  | 16.7 |  | 3 |  | 2.0 |
|  | 50-74 | 3 | 6 |  | 33.3 |  | 3 |  | 2.0 |  |  | dense | 3 | 0 |  | 0.0 |  | 0 |  | 0.0 |
|  | 75-100 | 4 | 1 |  | 5.6 |  | 1 |  | 0.7 |  |  |  |  |  |  |  |  |  |  |  |
| Right Middle | 0 | 0 | 2 |  | 11.1 |  | 108 |  | 72.0 |  | Right Middle | clear | 0 | 2 |  | 11.1 |  | 108 |  | 72.0 |
|  | <25 | 1 | 1 |  | 5.6 |  | 26 |  | 17.3 |  |  | hazy | 1 | 6 |  | 33.3 |  | 37 |  | 24.7 |
|  | 25-49 | 2 | 3 |  | 16.7 |  | 9 |  | 6.0 |  |  | moderate | 2 | 9 |  | 50.0 |  | 5 |  | 3.3 |
|  | 50-74 | 3 | 5 |  | 27.8 |  | 5 |  | 3.3 |  |  | dense | 3 | 1 |  | 5.6 |  | 0 |  | 0.0 |
|  | 75-100 | 4 | 7 |  | 38.9 |  | 2 |  | 1.3 |  |  |  |  |  |  |  |  |  |  |  |
| Right Lower | 0 | 0 | 2 |  | 11.1 |  | 96 |  | 64.0 |  | Right Lower | clear | 0 | 2 |  | 11.1 |  | 96 |  | 64.0 |
|  | <25 | 1 | 1 |  | 5.6 |  | 16 |  | 10.7 |  |  | hazy | 1 | 9 |  | 50.0 |  | 46 |  | 30.7 |
|  | 25-49 | 2 | 6 |  | 33.3 |  | 20 |  | 13.3 |  |  | moderate | 2 | 5 |  | 27.8 |  | 5 |  | 3.3 |
|  | 50-74 | 3 | 3 |  | 16.7 |  | 12 |  | 8.0 |  |  | dense | 3 | 2 |  | 11.1 |  | 3 |  | 2.0 |
|  | 75-100 | 4 | 6 |  | 33.3 |  | 6 |  | 4.0 |  |  |  |  |  |  |  |  |  |  |  |
| Left Upper | 0 | 0 | 7 |  | 38.9 |  | 131 |  | 87.3 |  | Left Upper | clear | 0 | 7 |  | 38.9 |  | 131 |  | 87.3 |
|  | <25 | 1 | 3 |  | 16.7 |  | 9 |  | 6.0 |  |  | hazy | 1 | 9 |  | 50.0 |  | 18 |  | 12.0 |
|  | 25-49 | 2 | 5 |  | 27.8 |  | 5 |  | 3.3 |  |  | moderate | 2 | 2 |  | 11.1 |  | 1 |  | 0.7 |
|  | 50-74 | 3 | 1 |  | 5.6 |  | 5 |  | 3.3 |  |  | dense | 3 | 0 |  | 0.0 |  | 0 |  | 0.0 |
|  | 75-100 | 4 | 2 |  | 11.1 |  | 0 |  | 0.0 |  |  |  |  |  |  |  |  |  |  |  |
| Left Middle | 0 | 0 | 5 |  | 27.8 |  | 113 |  | 75.3 |  | Left Middle | clear | 0 | 5 |  | 27.8 |  | 113 |  | 75.3 |
|  | <25 | 1 | 4 |  | 22.2 |  | 14 |  | 9.3 |  |  | hazy | 1 | 8 |  | 44.4 |  | 32 |  | 21.3 |
|  | 25-49 | 2 | 2 |  | 11.1 |  | 13 |  | 8.7 |  |  | moderate | 2 | 4 |  | 22.2 |  | 5 |  | 3.3 |
|  | 50-74 | 3 | 4 |  | 22.2 |  | 7 |  | 4.7 |  |  | dense | 3 | 1 |  | 5.6 |  | 0 |  | 0.0 |
|  | 75-100 | 4 | 3 |  | 16.7 |  | 3 |  | 2.0 |  |  |  |  |  |  |  |  |  |  |  |
| Left Lower | 0 | 0 | 0 |  | 0.0 |  | 104 |  | 69.3 |  | Left Lower | clear | 0 | 0 |  | 0.0 |  | 104 |  | 69.3 |
|  | <25 | 1 | 5 |  | 27.8 |  | 20 |  | 13.3 |  |  | hazy | 1 | 12 |  | 66.7 |  | 41 |  | 27.3 |
|  | 25-49 | 2 | 4 |  | 22.2 |  | 12 |  | 8.0 |  |  | moderate | 2 | 5 |  | 27.8 |  | 3 |  | 2.0 |
|  | 50-74 | 3 | 3 |  | 16.7 |  | 6 |  | 4.0 |  |  | dense | 3 | 1 |  | 5.6 |  | 2 |  | 1.3 |
|  | 75-100 | 4 | 6 |  | 33.3 |  | 8 |  | 5.3 |  |  |  |  |  |  |  |  |  |  |  |
